# Lipoic Acid Use and Functional Outcomes after Thrombolysis in Patients with Acute Ischemic Stroke and Diabetes

**DOI:** 10.1371/journal.pone.0163484

**Published:** 2016-09-27

**Authors:** Kang-Ho Choi, Man-Seok Park, Joon-Tae Kim, Hyung-Seok Kim, Ja-Hae Kim, Tai-Seung Nam, Seong-Min Choi, Seung-Han Lee, Byeong-Chae Kim, Myeong-Kyu Kim, Ki-Hyun Cho

**Affiliations:** 1 Department of Neurology, Chonnam National University Hwasun Hospital, Hwasun, Korea; 2 Department of Neurology, Chonnam National University Medical School, Gwangju, Korea; 3 Department of Forensic Medicine, Chonnam National University Medical School, Gwangju, Korea; 4 Department of Nuclear Medicine, Chonnam National University Medical School, Gwangju, Korea; Massachusetts General Hospital, UNITED STATES

## Abstract

**Background:**

Alpha-lipoic acid (aLA) is a strong antioxidant commonly used for treating diabetic polyneuropathy. Previously, we demonstrated the neurorestorative effects of aLA after cerebral ischemia in rats. However, its effects on patients with stroke remain unknown. We investigated whether patients treated with aLA have better functional outcomes after acute ischemic stroke (AIS) and reperfusion therapy than patients not receiving aLA.

**Methods:**

In this retrospective study of 172 prospectively registered patients with diabetes and AIS treated with tissue plasminogen activator (tPA), we investigated the relationship between aLA use and functional outcome both after 3 months and after 1 year. The functional outcomes included occurrence of hemorrhagic transformation (HT), early neurological deterioration (END), and early clinical improvement (ECI). Favorable outcomes were defined as modified Rankin Scale (mRS) scores of 0–2.

**Results:**

Of the 172 patients with AIS and diabetes, 47 (27.3%) used aLA. In the entire cohort, favorable outcomes occurred at significantly higher rates both at 3 months and at 1 year in those treated with aLA. The risks for END and HT were lower and the occurrence of ECI was higher in patients treated with aLA. In multivariable analysis, aLA use was associated with favorable outcomes both at 3 months and at 1 year. Age, HT, and increased National Institutes of Health Stroke Scale scores were negative predictors of a favorable outcome.

**Conclusions:**

The use of aLA in patients with AIS and diabetes who are treated with tPA is associated with favorable outcomes. These results indicate that aLA could be a useful intervention for the treatment of AIS after reperfusion therapy.

## Introduction

Despite significant advances in the prevention and treatment of stroke, it is still one of the leading causes of death and debilitating disease. Unfortunately, several neuroprotective strategies have failed in clinical trials. At present, it is reported that there are no pharmacological agents with putative neuroprotective actions that have demonstrated efficacy in improving outcomes after ischemic stroke in humans [1]. Therefore, there is a clear need for research to discover potential neuroprotective agents and new therapeutic strategies.

Previous stroke studies have confirmed that oxidative stress plays an important role in stroke and in reperfusion following stroke [2]. The ischemic brain is highly susceptible to oxidative damage due to its relatively low levels of protective antioxidants, its high levels of iron, which acts as a pro-oxidant under pathological conditions, its high concentrations of unsaturated lipids, and its high consumption of oxygen [3]. Paradoxically, the increased delivery of oxygen to ischemic brain tissue after reperfusion therapy may promote oxidative stress and cell death due to an increase in free radical generation [4]. Therefore, the use of antioxidants should be a promising strategy for treating ischemia-reperfusion injury.

Alpha-lipoic acid (aLA) is a strong antioxidant commonly used for the treatment of diabetic polyneuropathy (DPNP). We previously demonstrated the neuroprotective and neurorestorative effects of aLA, mediated at least partially via insulin receptor activation, after cerebral ischemia in rats [5]. Moreover, it has been reported that aLA is safe and significantly reduces oxidative stress levels in aged patients with diabetes mellitus complicated with acute ischemic stroke (AIS) [6].

To date, however, the effects of aLA on stroke outcome in patients with stroke and diabetes remain unknown. We investigated whether patients with diabetes treated with aLA have better functional outcomes after AIS and reperfusion therapy than patients not treated with aLA.

## Materials and Methods

### Subjects

This was a retrospective study of prospectively registered patients with AIS carried out using a single-center database. We used data from January 2006 until April 2014. Patients were consecutively enrolled if they 1) had AIS and were seen within 4.5 hours of symptom onset, 2) were treated with intravenous tissue plasminogen activator (IV-tPA), 3) had acute ischemic lesions on diffusion-weighted imaging (DWI), and 4) had diabetes mellitus with sensory symptoms. We excluded patients who 1) had previous symptomatic cerebral infarction, 2) had a previous modified Rankin Scale (mRS) score of 1 or more, 3) had an uncontrolled underlying medical disease, such as a malignant tumor, severe liver disease (Child-Pugh class B or more), or renal disease (creatine 3.0mg/dL or more), or 4) were treated with intra-arterial thrombolysis, as management patterns and devices have changed over the last 10 years.

Baseline clinical information was collected from all patients. Cerebrovascular risk factors noted are as follows: hypertension (previous use of antihypertensive medication, systolic blood pressure >140 mmHg, or diastolic blood pressure >90 mmHg at discharge), dyslipidemia (previous history of lipid-lowering medication), habitual smoking (current or past), and alcohol (>2 drinks or 20 g ethanol per day) [7].

### Ethics statement

This study was approved by the Institutional Review Board at the Chonnam National University Hospital. All of the clinical investigations described in this study were conducted in accordance with the principles expressed in the Declaration of Helsinki. Written informed consent was obtained from each patient or a family member.

### Clinical assessment and outcome measurements

Demographic characteristics, detailed histories, and clinical information regarding stroke risk factors were obtained. Physical examinations, routine laboratory tests, chest radiography, electrocardiography, and brain computed tomography were performed in all patients. The severity of the neurological deficits was assessed using the National Institutes of Health Stroke Scale (NIHSS) score, which is composed of 11 items [8]. In all items, a higher score indicates more severe impairment. The scale ranges from 0–42.

Functional outcomes 3 months and 1 year after the onset of symptoms were measured using mRS scores. The scores were categorized as favorable (mRS scores of 0–2) or unfavorable (mRS scores of 3–6) [9]. The mRS consists of 7 levels, ranging from perfect health without symptoms (mRS score 0) to death (mRS score 6). We defined early neurological deterioration (END) as an increase of 1 or more points in motor power or an increase of 2 or more points in total NIHSS score. Early clinical improvement (ECI) was defined as a decrease of 4 or more points in the NIHSS score within 7 days [10, 11]. Hemorrhagic transformation (HT) was considered present when one or more of the follow-up gradient echo magnetic resonance imaging (MRI) or CT scans showed a region consistent with an acute infiltration of blood. Patients underwent MRI on admission and at day 5. They also underwent CT or an MRI when there was a worsening of symptoms. Based on clinical and neuro-imaging findings, the patients were classified based on stroke subtypes according to the Trial of Org. 10172 in Acute Stroke Treatment (TOAST) classification. There were 5 subtypes: 1) large-artery atherosclerosis (LAA), 2) cardioembolism (CE), 3) small vessel occlusion (SVO), 4) stroke of undetermined etiology (UD), and 5) stroke of other determined etiology (OE) [12].

**Fig 1:**
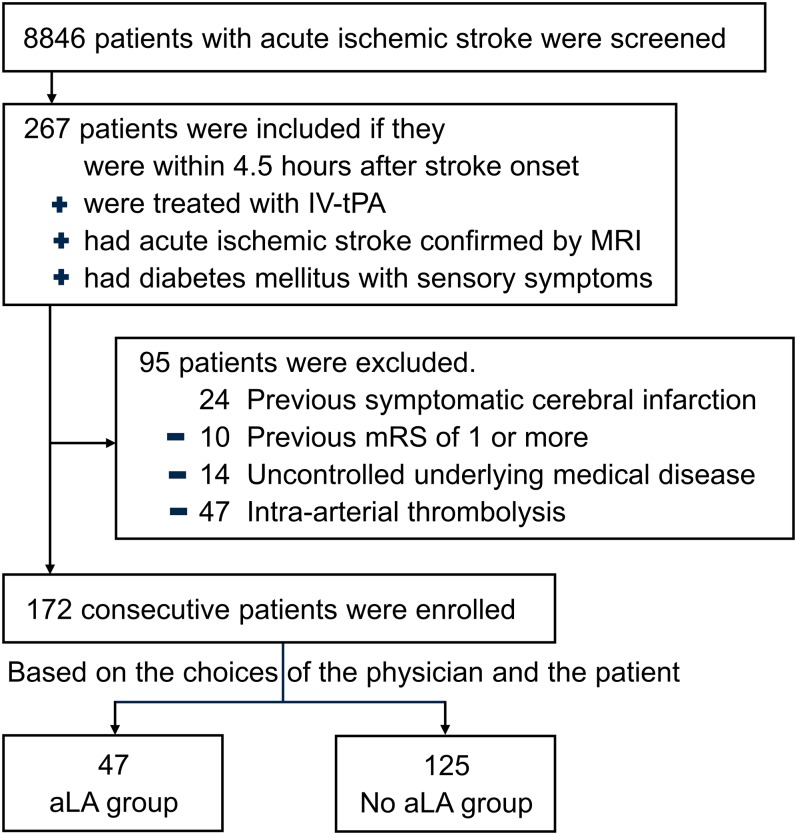
The flow diagram shows the process of grouping of subjects from screening to the completion of the study.

We provided the appropriate treatment depending on best practice guidelines established by the American Heart Association and the American Stroke Association for all patients in this study [1, 13]. The protocol for therapy of diabetes encouraged, but did not mandate, the use of drug classes with evidence for reduction of diabetic complications, such as DPNP. These drugs include aLA, pregabalin, gabapentin, venlafaxine, duloxetine, and tricyclic antidepressants for patients with diabetes [14]. The use of the specific drug class was based on physicians’ attitudes regarding the protocol. In addition, aLA was considered for patients who were willing to take the medicine before meals, as the medication should be taken before meals. In the aLA group, aLA was administered after overnight fasting within 24 hours of symptom onset based on the choices of the physician and the patient (Fig 1). Treatment was continued for at least the first three months after AIS. The aLA dose was 600 mg once daily.

### Statistical analysis

Differences between the groups were analyzed using Student’s *t* test, one-way ANOVA, or Mann–Whitney test where appropriate. The chi-square test was used for non-continuous variables. Spearman’s and Pearson’s correlation tests were carried out to reveal any correlations. The level of significance was set at a *p* value of <0.05.

The Cochran–Mantel–Haenszel (CMH) test carried out across the seven-level mRS score for shift analysis and conventional dichotomized analysis were used to analyze the distributions of the 3-months and 1-year mRS outcome scales [15]. We performed a backward stepwise logistic regression analysis to assess the odds ratios (ORs) and the corresponding 95% confidence intervals (CIs) for a favorable outcome according to the use of aLA. We used two multivariable models. Model 1 was initially adjusted for age and gender. Potential factors already established as predictors of stroke outcome were added to model 2.

Variables that were not significant (*p* >0.1) were sequentially removed from the full model. The excluded potential confounders were reintroduced at various stages of model development until only the significant independent variables remained. A two-sided p-value of <0.05 was considered statistically significant. All statistical analyses were performed using PASW software (version 18.0).

## Results

### Patient characteristics

The study included 172 patients with AIS and diabetes who were treated using thrombolysis. Of the included patients, 47 (27.3%) were using aLA at the acute stage of stroke. Baseline clinical and biochemical characteristics of the aLA users and the nonusers are shown in Table 1. Vascular risk factors, biochemical variables, and stroke subtypes were not significantly different between the two groups. There was also no difference in the initial NIHSS score or the onset to intravenous thrombolysis (IVT) time.

**Table 1:** Baseline differences in clinical and biochemical characteristics according to the use of alpha-lipoic acid.

	aLA (n = 47)	No aLA (n = 125)	*p* value*
Age, years	68.6	71.0	0.169
Male, **n (%)**	27 (57.4)	62 (49.6)	0.362
**Risk factors, n (%)**			
Hypertension	34 (72.3)	99 (79.2)	0.341
Atrial fibrillation	17 (36.2)	43 (34.4)	0.829
Dyslipidemia	8 (17.0)	16 (12.8)	0.185
Coronary angioplasty	1 (2.1)	1 (0.8)	0.407
Smoking	15 (31.9)	35 (28.0)	0.617
Alcohol	15 (31.9)	40 (32.0)	0.398
**Biochemical variables (mean ± SD)**			
Total-C, mg/dL	173.1± 41.4	176.7 ± 39.7	0.657
LDL-C, mg/dL	113.7± 35.7	113.5 ± 35.0	0.998
Triglyceride, mg/dL	102.4 ± 61.1	107.9 ± 53.0	0.604
HDL-C, mg/dL	44.3 ± 12.0	45.1 ± 16.4	0.789
Creatinine, mg/dL	0.8 ± 0.4	0.9 ± 0.3	0.680
Glycated hemoglobin, %	7.1 ± 1.9	7.3 ± 1.3	0.419
FBS, mg/dL	165.5 ± 72.4	179.0 ± 72.8	0.290
**TOAST classification, n (%)**			0.922
LAA	17 (36.2)	42 (33.6)	
CE	14 (29.8)	41 (32.8)	
SVO	3 (6.4)	6 (4.8)	
UD	12 (25.5)	35 (28.0)	
OE	1 (2.1)	1 (0.8)	
Initial NIHSS score (mean ± SD)	9.5 ± 4.9	10.5 ± 4.9	0.149
Baseline mRS (mean ± SD)	3.3 ± 1.2	3.5 ± 1.3	0.443
Onset to IVT time (mean ± SD), min.	123.0 ± 67.8	132.1 ± 63.9	0.429
Discharge medication, n (%)			
Statin	32 (68.1)	89 (71.8)	0.638
Antihypertensive drug	33 (70.2)	97 (77.6)	0.318

*Continuous variables were compared between groups using Student’s *t* tests, one-way ANOVAs, or Mann–Whitney tests. The chi-square test was used for non-continuous variables.

Total-C = total cholesterol; LDL-C = low-density lipoprotein cholesterol; HDL-C = high-density lipoprotein cholesterol; FBS = fasting blood sugar; TOAST = Trial of Organization 10172 in Acute Stroke Treatment; LAA = large-artery atherosclerosis; CE = cardioembolism; SVO = small-vessel occlusion; UD = stroke of undetermined etiology; OD = stroke of other determined etiology; NIHSS = National Institutes of Health Stroke Scale; SD = standard deviation; mRS = modified Rankin Scale; IVT = intravenous thrombolysis

### Functional Outcomes

**Fig 2:**
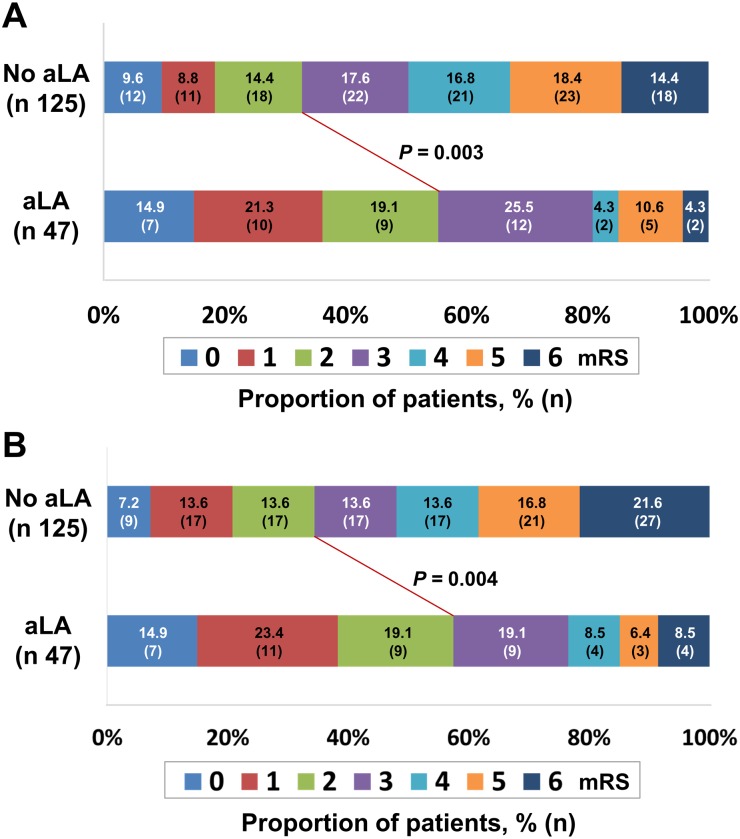
Distribution of the modified Rankin Scale (mRS) scores among patients classified based on the use of alpha-lipoic acid (aLA) 3 months (A) and 1 year (B) after ischemic stroke. The lines indicate differences in mRS categories (mRS scores of 0–2 vs. 3–6) between groups classified by aLA use. The *p*-value refers to the significance level of the chi-square test used to compare proportions.

**Fig 3:**
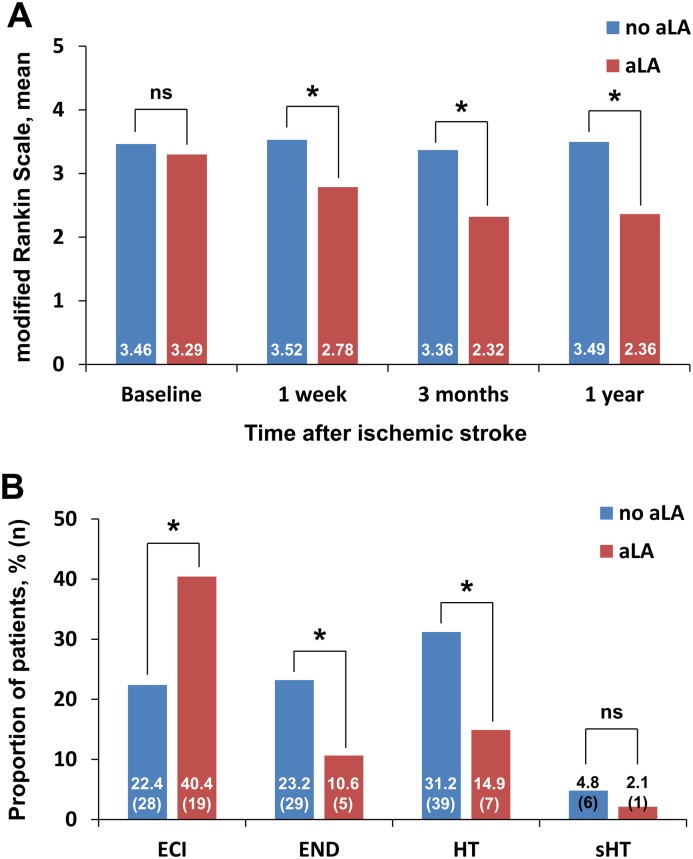
(A) Changes from baseline modified Rankin Scale (mRS) up to 1 year after stroke. (B) Proportion of patients with early clinical outcomes and hemorrhagic transformation displayed according to the use of alpha-lipoic acid (aLA). *p < 0.01 vs. no-aLA group; the chi-square test was used to compare proportions. ECI = early clinical improvement; END = early neurological deterioration; HT = hemorrhagic transformation; sHT = symptomatic hemorrhagic transformation.

After 3 months, 67 patients (38.9%) had a favorable outcome (mRS score of 0–2). As shown in Fig 2, the number of patients with a favorable outcome was significantly higher in the aLA user group compared to the aLA non-user group (55.3 vs. 32.8%, *p* = 0.003; Fig 2A). Furthermore, the percentage of patients who were living without a significant disability as measured by mRS assessed 1 year after ischemic stroke (40.7% in total) was significantly higher in the group treated with aLA (57.4% versus 34.4%, *p* = 0.004; Fig 2B). Next, we confirmed the above results using the CMH test across the seven-level mRS scores for shift analysis. The CMH test revealed a statistically significant shift in the 3-months and 1-year mRS distributions favoring aLA (*p* < 0.001). Changes from baseline mRS up to 1 year after stroke showed favorable outcomes by the treatment of aLA (Fig 3A).

Univariate analyses of variables associated with functional outcomes are shown in Table 2. Age, atrial fibrillation, initial NIHSS score, and HT were significantly associated with functional outcomes at 3 months and 1 year as determined by univariate analysis.

In the final multivariable analysis, which included adjustments for confounders, such as age, sex, history of hypertension, atrial fibrillation, dyslipidemia, HT, and initial NIHSS score, aLA use was significantly associated with a favorable outcome 3 months after ischemic stroke (OR = 2.13, 95% CI [1.01, 4.51], *p* = 0.048; Table 3). Similarly, aLA use was still associated with a favorable outcome 1 year after ischemic stroke (OR = 2.26, 95% CI [1.06, 4.84], *p* = 0.036; Table 3). Age, HT, and increasing NIHSS scores were negative predictors of favorable outcomes after adjusting for all potential factors (Table 3). The following values were selected using multiple threshold estimations: age (≥70), and NIHSS score (≥8).

**Table 2:** Variables related to functional outcomes at 3 months and 1 year used in univariate analyses.

	3-month outcomes			1-year outcomes		
	Patients with mRS scores of 0–2 (n = 67)	Patients with mRS scores >2 (n = 105)	*p* value*	Patients with mRS scores of 0–2 (n = 70)	Patients with mRS scores >2 (n = 102)	*p* value*
Age, years	66.5	72.7	<0.001	65.7	73.5	<0.001
Male, n (%)	41 (61.2)	48 (45.7)	0.060	42 (60.0)	47 (46.1)	0.088
aLA use, n (%)	26 (38.8)	21 (20.0)	0.009	27 (38.6)	20 (19.6)	0.009
**Risk factors, n (%)**						
Hypertension	52 (77.6)	81 (77.1)	1.000	55 (78.6)	78 (76.5)	0.853
Atrial fibrillation	17 (25.4)	43 (41.0)	0.049	18 (25.7)	42 (41.2)	0.049
Dyslipidemia	9 (13.4)	15 (14.3)	0.809	9 (12.8)	15 (14.7)	0.564
Coronary angioplasty	1 (1.5)	1 (0.9)	0.407	1 (1.5)	1 (0.9)	0.407
Smoking	21 (31.3)	29 (27.6)	0.610	22 (31.4)	28 (27.5)	0.610
Alcohol	20 (29.8)	35 (33.3)	0.398	21 (30.0)	34 (33.3)	0.319
**Biochemical variables (mean ± SD)**						
Total-C, mg/dL	178.4 ± 43.7	173.9 ± 45.9	0.527	177.4 ± 39.1	174.5 ± 48.8	0.679
LDL-C, mg/dL	116.5 ± 43.6	111.6 ± 43.2	0.493	114.1 ± 39.4	113.3 ± 46.0	0.908
Triglyceride, mg/dL	102.5 ± 54.5	109.1 ± 65.1	0.496	101.8 ± 53.5	109.8 ± 65.9	0.406
HDL-C, mg/dL	45.6 ± 12.8	44.3 ± 14.2	0.573	46.7 ± 13.3	43.5 ± 13.7	0.141
Creatinine, mg/dL	0.8 ± 0.3	0.9 ± 0.6	0.155	0.8 ± 0.4	0.9 ± 0.6	0.123
Glycated hemoglobin, %	7.3 ± 1.5	7.2 ± 1.5	0.682	7.3 ± 1.5	7.2 ± 1.5	0.781
FBS, mg/dL	172.7 ± 72.1	177.2 ± 72.9	0.702	170.2 ± 71.7	178.9 ± 73.0	0.448
Initial NIHSS (mean ± SD)	8.1 ± 3.5	11.7 ± 4.5	<0.001	8.1 ± 3.6	11.8 ± 4.4	<0.001
Onset to IVT (mean ± SD), min.	124.8 ± 63.2	132.5 ± 66.2	0.468	122.2 ± 65.1	134.6 ± 64.7	0.237
TOAST classification, n (%)^†^	17 (25.4)	38 (36.2)	0.180	18 (25.7)	37 (36.3)	0.183
Hemorrhagic transformation, n (%)	8 (11.9)	38 (36.2)	<0.001	10 (14.3)	36 (35.3)	0.003

*Continuous variables were compared between the groups using Student’s *t* tests, one-way ANOVAs, or Mann–Whitney tests. The chi-square test was used for non-continuous variables.

^†^Percentage relates to cardiac embolism, because this cause was associated with a worse outcome.

Total-C = total cholesterol; LDL-C = low-density lipoprotein cholesterol; HDL-C = high-density lipoprotein cholesterol; FBS = fasting blood sugar; NIHSS = National Institutes of Health Stroke Scale; IVT = intravenous thrombolysis; TOAST = Trial of Organization 10172 in Acute Stroke Treatment; aLA = alpha-lipoic acid; SD = standard deviation

**Table 3:** Final logistic regression model with predictors of favorable outcome*.

Independent Variables	Outcomes (hazard ratio [95% CI])			
	3-month outcomes		1-year outcomes	
	Model 1	Model 2	Model 1	Model 2
aLA use	2.687 [1.014, 4.819]	2.134 [1.008, 4.517]	2.817 [1.074, 4.897]	2.258 [1.055, 4.835]
Age (≥70)	0.468 [0.243, 0.902]	0.493 [0.253, 0.958]	0.306 [0.157, 0.596]	0.317 [0.161, 0.622]
Male	0.543 [0.219, 1.131]	0.588 [0.261, 1.194]	0.631 [0.287, 1.912]	0.710 [0.329, 1.530]
Hypertension	1.052 [0.485, 2.283]	1.139 [0.505, 2.568]	1.162 [0.526, 2.570]	1.256 [0.551, 2.864]
Atrial fibrillation	0.607 [0.300, 1.227]	0.674 [0.315, 1.445]	0.637 [0.313, 1.298]	0.671 [0.313, 1.442]
Dyslipidemia	0.934 [0.339, 2.573]	1.318 [0.440, 3.955]	1.148 [0.415, 3.175]	1.550 [0.522, 4.602]
Hemorrhagic transformation	0.243 [0.100, 0.589]	0.283 [0.113, 0.710]	0.343 [0.146, 0.802]	0.403 [0.165, 0.982]
Initial NIHSS score (≥8)	0.256 [0.127, 0.516]	0.266 [0.129, 0.546]	0.264 [0.129, 0.541]	0.269 [0.129, 0.565]

* Model 1 was adjusted for age and gender. Model 2 was adjusted for the variables in model 1 plus factors already established as predictors of stroke outcome and differed significantly between the outcome groups in univariate analysis. CI = confidence interval; NIHSS = National Institutes of Health Stroke Scale

### Early clinical outcomes

Forty-seven (27.3%) patients had ECI. END occurred in 34 (19.7%) cases. ECI was more prevalent in aLA users (40.4% vs. 22.4%, *p* = 0.030), whereas END was significantly less prevalent in aLA users compared to patients not using aLA (10.6% vs. 23.2%, *p* = 0.036; Fig 3B). In the entire cohort, HT was more often seen in patients not receiving aLA compared to patients using aLA (31.2% vs. 14.9%, *p* = 0.017). However, the frequency of symptomatic HT was comparable between the two groups (4.8% vs. 2.1%, *p* = 0.432; Fig 3B).

## Discussion

This is the first study to evaluate the effects of aLA in a cohort of patients with diabetes and acute stroke treated with a thrombolytic agent. This study showed that patients with diabetes treated with aLA have better functional outcomes following AIS after reperfusion therapy than patients not using aLA. Previously, we demonstrated the neuroprotective and neurorestorative effects of aLA after cerebral ischemia in rats subjected to middle cerebral artery occlusion [5]. The results of the current study indicate that aLA use after IVT is independently associated with favorable outcomes (mRS scores of 0–2) 3 months and 1 year after ischemic stroke. Our study suggests that patients treated with aLA may have better long-term functional outcomes than patients not using aLA. The findings highlight the importance of antioxidants in patients treated with thrombolysis to improve the outcome of AIS. Given the numerous failures in the clinical translation of neuroprotective treatment for patients with AIS, the current results are of potential importance in the clinical setting.

The mechanisms by which aLA provides benefits to patients with AIS may be related to the antioxidant properties of aLA. Oxidative stress is one of the leading causes of brain damage in ischemia-reperfusion injury [2], and aLA is a strong antioxidant that influences a number of cellular processes, including the scavenging of reactive oxygen species (ROS), the regeneration of reduced forms of other antioxidants, and the modulation of transcription factor activity. It has been shown that aLA is able to repair oxidative damage, regenerate endogenous antioxidants, improve endothelial function and blood flow, and accelerate glutathione synthesis, which plays a crucial role in preventing damage caused by ROS to important cellular components [16–19].

In addition, the possible mechanism by which aLA promotes favorable outcomes may be related to activation of the insulin receptor (IR). In our previous study, the effects of aLA were significantly associated with IR activation, which is a well-known neuroprotective pathway in ischemic models [5, 20–22]. aLA has been reported to be safe and effective in the treatment of aged patients with diabetes mellitus complicated by AIS. It has been shown to significantly reduce the patient's oxidative stress and blood glucose and lipid levels, and to improve islet function in a previous study [6]. Because only diabetic patients were enrolled in our study, aLA may appear to be more useful in improving ischemic stroke than in other studies that include non-diabetic patients.

A previous study has shown that aLA has a stabilizing effect on the blood-brain barrier (BBB), making aLA an attractive therapeutic agent for the treatment of stroke [23]. BBB damage is a common event in ischemic stroke and is aggravated by reperfusion [24, 25]. Therefore, one of the most important targets for the effective treatment of cerebral ischemia is the protection of the BBB against damage [26]. HT, one of the most serious complications of ischemic stroke, is induced by the disruption of the BBB after ischemia-reperfusion [25]. In our study, HT was a significant independent negative predictor of favorable outcome after AIS in patients treated using thrombolysis. Importantly, HT was less prevalent in aLA users compared to patients not using aLA. This may be a major mechanism underlying the positive effects of aLA.

In addition to HT, age and increased NIHSS scores have already been established as negative predictors of favorable outcomes. These factors remained negative predictors of favorable outcomes after adjusting for all potential factors. These findings are similar to those of previous reports of outcome measures in patients treated with thrombolytics [27, 28].

This study has limitations. First, it was based on a nonrandomized prospective registry with a relatively small number of patients. Therefore, there is a risk of bias from unmeasured or residual confounders despite adjustments for covariates. Although this was a retrospective study, we prospectively collected data in consecutive patients. Second, recanalization was not analyzed in this study and could affect the prognosis. Because we performed non-contrast CT-based IV thrombolysis, we could not confirm recanalization in the patients. Finally, data from the literature indicate that the effects of aLA depend on the dose used [29]. However, we cannot be sure of the doses and the durations of the aLA pretreatment. It is thus possible that the beneficial effects depend on the duration of treatment before stroke. However, there was no withdrawal of aLA in the acute phase of stroke or after hospitalization.

In conclusion, we show, for the first time, a beneficial effect of aLA on ischemia-reperfusion injury in patients with diabetes and AIS. These results indicate that aLA could be a useful intervention for the treatment of AIS after reperfusion therapy. Despite the acknowledged limitations of our study, this analysis may have implications for future multicenter randomized trials. One such trial is currently being performed and will be the subject of a following study (IMpact of liPOic acid use on stRoke ouTcome After thrombolysis in patieNts with diabeTes [IMPORTANT]; Honam Research Council of Stroke of Korea).
